# Air pollution and postpartum depression: the interplay with prenatal stress

**DOI:** 10.21203/rs.3.rs-8523355/v1

**Published:** 2026-02-02

**Authors:** Gary Joseph, Megan Niedzwiecki, Itai Kloog, Allan C. Just, Ivan. Gutierrez-Avila, Elena Colicino, Martha María Téllez-Rojo, Robert O. Wright, Carmen Hernandez-Chavez, Gabriela Gil Martínez, Rosalind J. Wright, Veerle Bergink, Lauren M. Petrick

**Affiliations:** Icahn School of Medicine at Mount Sinai; Icahn School of Medicine at Mount Sinai; Icahn School of Medicine at Mount Sinai; Brown University School of Public Health; Icahn School of Medicine at Mount Sinai; Icahn School of Medicine at Mount Sinai; National Institute of Public Health; Icahn School of Medicine at Mount Sinai; National Institute of Perinatology; National Institute of Perinatology; Icahn School of Medicine at Mount Sinai; Icahn School of Medicine at Mount Sinai; Icahn School of Medicine at Mount Sinai

**Keywords:** Air pollution, postpartum depression, prenatal stress

## Abstract

**Background:**

Postpartum depression (PPD) is a global health issue that can lead to high levels of maternal morbidity. We previously found that ambient air pollution (PM_2.5_) during pregnancy is associated with PPD. However, the modifying role of psychosocial stress on this relationship is unclear.

**Methods:**

We measured pregnancy stress in the PROGRESS cohort (n = 475 mothers) using negative life events (NLE) and perceived stress score (PSS). We assessed the modifying role of NLE and PSS on the association between prenatal PM_2.5_ exposure and PPD at 6 months. Daily residence level PM_2.5_ estimates generated from a spatiotemporal model was averaged over pregnancy. PPD was assessed using the Edinburgh Postnatal Depression Scale (EPDS ≥ 13) and categorized as chronic depression (EPDS ≥ 13 during pregnancy and at 6 months), new-onset PPD (EPDS < 13 during pregnancy and EPDS ≥ 13 at 6 months), or prevalent PPD (EPDS ≥ 13 at 6 months, regardless of EPDS score during pregnancy). Modified Poisson regression evaluated the association between PM_2.5_ and PPD, stratified by NLE and PSS scores, dichotomized around the median (low/high). We repeated the analyses with other proposed EPDS cut-offs for Mexico (EPDS ≥ 10 and ≥ 12).

**Results:**

Each 5-μg/m^3^ increase in average pregnancy PM_2.5_ exposure was associated with 129% higher risk of prevalent PPD among mothers with low PSS (RR: 2.29, 95% CI: 1.17–4.47). The risk of new-onset PPD at 6 months per 5-μg/m^3^ increase in PM_2.5_ during pregnancy quadrupled among mothers with low PSS (RR: 4.58, 95% CI: 1.83–11.49) and high NLE (RR: 4.71, 95% CI: 1.72–12.92) scores. Similar findings were observed with an EPDS ≥ 10 or EPDS ≥ 12 cut-off.

**Conclusion:**

The risk of PPD from PM_2.5_ exposure was enhanced in mothers with low PSS or high NLE scores, especially new-onset PPD, regardless of the EPDS threshold used. These findings suggest that well-characterized stress phenotyping during pregnancy may help identify which women are at-risk for depression.

## Introduction

Stress is a biological and physiological response to challenges and threats (1). Although stress is a normal part of life, its intensity and frequency can heighten during pregnancy due to physiological and psychological changes (2, 3) and worries related to pregnancy and parenting (i.e., labor and delivery, financial constraints) (4). Thus, stress can act as an exposure capable of triggering biological response including neuroendocrine and immune changes (5). During pregnancy, the maternal endocrine, nervous, and immune systems undergo adjustments to support fetal development and maternal health (6). However, prenatal stress can disrupt these processes through dysregulation of the hypothalamic-pituitary-adrenal (HPA) axis (6), increase corticotropin-releasing hormone (CRH) (6, 7) and inflammatory cytokines (6, 8), resulting in downstream health effects in the mother (6, 9, 10).

Prenatal stress is associated with various maternal health outcomes (9, 10), including postpartum depression (PPD) (6, 11, 12). Findings from a recent systematic review and meta-analysis with 17 cohort studies showed a 82% increase in PPD in women exposed to a prenatal stressful life event (13). Further, studies suggest the role of HPA axis dysregulation including dysfunction in synaptic transmission and the neurotransmitter system in the mechanism that links stress to PPD (14–17). While emerging studies point to additional environmental exposures that can impact the same HPA axis and neuroendocrine pathways to exacerbate or antagonize these associations, most studies have only looked at stress as a single exposure (13, 18–20).

Ambient air pollution (PM_2.5_) is an exposure positively associated with PPD, (21) which may act on similar mechanisms as stress (22, 23). There was a 56% increase in PPD risk among mothers with higher PM_2.5_ exposure during the second trimester of pregnancy (22), similar to a study conducted by our team which found a 59% increased risk of PPD per 5-μg/m3 increase in average PM_2.5_ exposure during pregnancy (24). However, findings from a recent meta-analysis of 12 European birth cohorts found no association between PM_2.5_ and PPD. While variability in PM_2.5_ and PPD assessment methods between the cohorts, heterogeneity across the cohorts, and bias from complete case analysis likely influenced the analysis (25), null findings in the meta-analysis may also suggest additional confounders.

Because both prenatal stress and PM_2.5_ are individually associated with PPD, pregnant women may be particularly more vulnerable to their combined effects, as these stressors may interact through shared biological pathways to exacerbate the risk of PPD. However, while a study in mice suggests that maternal stress exposure during late gestation heightens offspring susceptibility—particularly in males—to the harmful effects of prenatal air pollutant exposure on neurocognitive disorders in adulthood (26), research that examined the interplay of prenatal stress on the association between PM_2.5_ and the risk of PPD in humans remains limited. Further, while the Edinburgh Postnatal Depression Scale (EPDS) is a commonly used screening tool for identifying probable PPD, the use of varying cut-off scores such as 7/8 (27), 9/10 (28), 11/12 (27), ≥ 12 (29, 30), and ≥ 14 (31) in Mexico, can complicate cross-study comparisons and interpretations. For example, our team previously used an EPDS threshold of 13 or more (24, 32) in analyses from the Programming Research in Obesity, Growth, Environment, and Social Stressors (PROGRESS) cohort as a conservative threshold to reduce the probability of false positives within the context of a population-based cohort allowing for the identification of cases with more marked symptomatology.

In this study, we assessed the role of prenatal stress—negative life events (NLE) and perceived stress on the association between PM_2.5_ and PPD using data from a prospective cohort of Mexican mothers. Understanding their potential joint effects is critical for identifying vulnerable women and informing preventive screening strategies. We primarily used an EPDS cut-off score of ≥ 13, consistent with the threshold commonly applied in previous PROGRESS studies and assessed the robustness of our findings using two of the proposed cut-offs (≥ 10 and ≥ 12) for screening probable PPD in Mexico.

## Methods

### Study population

Healthy pregnant women who received prenatal care through the Mexican Social Security Institute (Instituto Mexicano del Seguro Social – IMSS) between 2007 and 2011 were recruited in the PROGRESS study (24). Participants were included in the study if they met the following criteria: less than 20 weeks of gestation, singleton pregnancy, at least 18 years old, completed primary education, intended to remain in Mexico City for the next three years, had telephone access, no history of heart or kidney disease, did not consume alcohol daily, reported no drug addiction, and were not taking steroids or anti-epileptic medications. This study included 475 participants with complete data on PPD at 6 months postpartum, prenatal stress, pregnancy PM_2.5_, and all covariates. The sample size in this study is smaller than that of the original cohort study on PM_2.5_ PPD (509 participants) (24) due to missing data on perceived stress for approximately 7% of the participants.

### Outcomes

The Spanish version of the EPDS, validated in Mexico (29, 31), was used to collect data on mother’s depressive symptoms during the second or third trimester of pregnancy and at 6 months postpartum. EPDS is a widely used screening tool to identify and monitor postpartum depressive symptoms in mothers (33–35). The EPDS is a 10-item self-report questionnaire that assesses how the participants felt over the past seven days (36). These items include: “I have laughed and been able to see the funny side of things,” “I have looked forward with enjoyment to things,” “I have blamed myself unnecessarily when things went wrong,” “I have been anxious or worried for no good reason,” “I have felt scared or panicky for no very good reason,” “Things have been getting on top of me,” “I have been so unhappy that I have had difficulty sleeping,” “I have felt sad or miserable,” “I have been so unhappy that I have been crying,” and “The thought of harming myself has occurred to me.” Participant’s responses are scored on a 4-point Likert scale, from zero (0) to three based on the seriousness of the symptom. A score of zero (0) indicates that the participant is the most favorable condition (i.e., unlikely to have a clinically significant depressive symptom), while a score of three suggests the least favorable condition (i.e., likely to have a clinically significant depressive symptom). The total score, ranging from zero (0) to 30 is calculated by summing the scores of all 10 items.

We applied the commonly used threshold in PROGRESS publications (EPDS ≥ 13: Yes/No) to identify women with probable PPD, a clinically relevant cut-off used in other settings to identify women at risk for major depression (36, 37). Because lower cut-offs have been proposed for screening women with probable PDD in Mexico (27–31), we compared our findings with other cut-off values of EPDS ≥ 10 and EPDS ≥ 12. These cut-off values refer to the most updated thresholds proposed for screening for major depressive symptoms in adult women in Mexico (28, 30). This strategy allowed us to assess whether the observed associations between PM_2.5_ and PPD are consistent, and not sensitive to a specific threshold.

We assessed prevalent PPD, chronic PPD, and new-onset PPD at 6 months using the three cut-off values. Chronic PPD included mothers with an EPDS ≥ 13 during pregnancy and at 6 months postpartum. New-onset PPD included mothers with an EPDS < 13 during pregnancy but EPDS ≥ 13 at 6 months postpartum (24). Prevalent PPD refers to any mother with an EPDS ≥ 13 at 6 months postpartum, regardless of EPDS score during pregnancy (24). Thus, prevalent depression included mothers with both chronic PPD, and new-onset PPD. Similar definitions were applied when considering the EPDS ≥ 10 and EPDS ≥ 12 cut-offs.

### Prenatal PM_2.5_ exposure during pregnancy

Daily PM_2.5_ exposure for each participant’s residence was assessed during pregnancy using a validated spatiotemporal model (38). The model integrated data from Moderate Resolution Imaging Spectroradiometer (MODIS) satellite-derived Aerosol Optical Depth (AOD) at a 1×1 km spatial resolution with ground-based PM_2.5_ measurements, meteorological data (including temperature, relative humidity, wind speed, and planetary boundary layer height), and land use regression variables (such as roadway density). Mixed-effects modeling, which incorporated spatial and temporal predictors alongside day-specific random effects, was employed to capture temporal variability in the relationship between PM_2.5_ and AOD. The model incorporated a seasonal smoothing function for latitude and longitude, along with a time-varying average, to integrate local monitoring data on days when AOD measurements were unavailable. The model exhibited strong predictive accuracy, with an R^2^ of 0.724 from cross-validation, and has undergone external validation in prior studies (39, 40). Daily exposure estimates were assigned to participants based on GPS coordinates of their residential locations, collected by study personnel. Gestational age was determined using maternal reports of the last menstrual period and standardized physical examinations. The average PM_2.5_ exposure during pregnancy was calculated using the average PM_2.5_ measurements from each trimester (1–13 weeks for the first trimester, 14–27 weeks for the second, and 28 weeks to delivery for the third). We convert PM_2.5_ exposure to a 5-unit change during pregnancy to simplify the interpretation of its effects on the outcomes. More details on the model’s development and validation are available elsewhere (38).

### Prenatal stress during pregnancy.

NLE was measured using the Crisis in Family Systems-Revised (CRISYS) survey, previously validated in Spanish (41) CRISYS is a flexible, multidimensional, and standardized tool developed to quantify contemporary sources of life stress (42). It measures life events across 11 domains: financial, legal, career, relationship, home safety, neighborhood safety, medical issues (self and others), home, prejudice, and authority (42). Participants in their third trimester of pregnancy(43) indicated whether they experienced specific life events in the past six months as positive, negative or neutral. Therefore, the NLE captures stress during the pregnancy period. The NLE domain score is calculated by summing the number of domains that have one or more negative events. NLE score ranges from 0 to 11, with higher score indicating greater stress (42). Perceived stress score (PSS) during the third trimester of pregnancy was measured using the validated Spanish version of the 4-item Perceived Stress Scale (PSS-4) (44, 45). Participants rated their perceived stress level in a 5-point Likert scale, ranging from zero (0) to 4 (46). The PSS score is calculated by summing the scores of individual items, with a higher score indicating greater perceived stress (44).

### Covariates

Maternal age (in years), maternal level of education (less than high school, high school, more than high school), socioeconomic status (SES), body mass index (BMI) pre-pregnancy, maternal exposure to smoke inside the home (yes/no), prenatal stress, and gestational age at birth (in weeks) were used as covariates in this study. SES in PROGRESS is calculated based on the Mexican Association of Market and Public Opinion Research Agencies (Spanish acronym AMAI) (47). We used maternal exposure to second-hand smoke inside the home instead of self-reported smoking during pregnancy, as only one mother reported smoking during pregnancy.

#### Statistical analysis

We performed descriptive analysis for all the variables included in this study. We assessed the association between mean PM_2.5_ during pregnancy and the outcomes using modified Poisson regression with robust error variance, allowing us to calculate the relative risk for the current study sample size (24). To evaluate departures from multiplicativity, we tested for interactions by including a product term between the average PM_2.5_ and prenatal stress (NLE and PSS levels) dichotomized around the median (48, 49). We then stratified the model by low and high stress levels. The analyses were adjusted for maternal age, maternal education, maternal exposure to smoke inside the home, and gestational age at birth. Statistical significance was determined at a p-value of less than 0.05. All the analyses were conducted using R, version 4.4.1 (2024-06-14 ucrt).

## Results

Table 1 summarizes the characteristics of the study population. On average, maternal age during pregnancy was 27.7 (SD: 5.5). PM_2.5_ exposure during pregnancy was 22.9 μg/m^3^ (IQR: 20.3–24.5). Maternal education ranged from 23.4% among mothers with more than a high school education to 41.1% among those with less than a high school education. Approximately 11% of mothers belonged to a high socioeconomic status, while 51.4% were classified as low socioeconomic status. The median NLE and PSS scores during pregnancy were 3 (IQR: 2–5) and 5 (IQR: 3–7), respectively. At 6 months postpartum, median EPDS score was 5 (IQR: 2–10). Over 18% of mothers had prevalent PPD, 9.7% had chronic PPD, and 8.6% had new-onset PPD, using a cut-off of EPDS ≥ 13. However, considering an EPDS ≥ 10, 30.3% of mothers identified with prevalent depression, 20.8% with chronic PPD, and 9.5% with new-onset PPD (Table 1).

**Figure 1:**
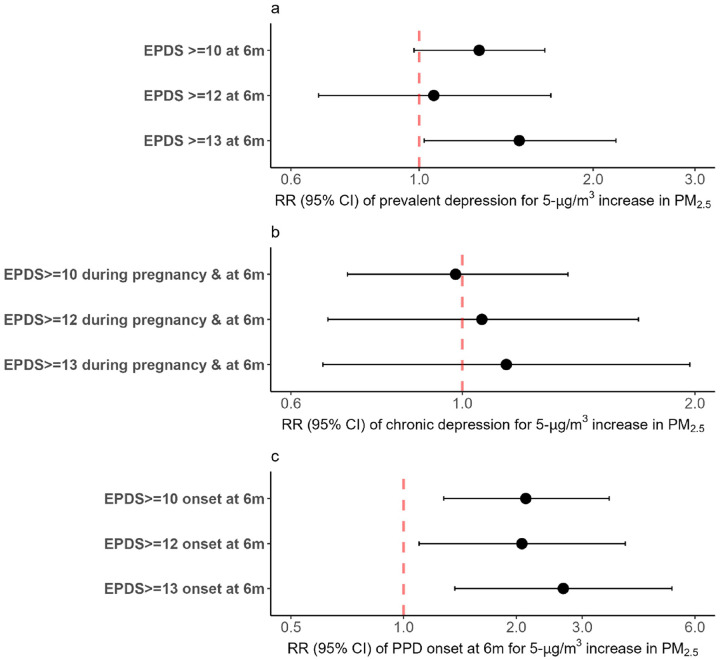
Association between the average particulate air pollution (PM_2.5_) exposure during pregnancy and postpartum depression (prevalent, new-onset, and chronic depression). Analyses were adjusted for maternal age, maternal education, socioeconomic status, body mass index, maternal exposure to smoke inside the home, and gestational age at birth. EPDS: Edinburgh Postnatal Depression Scale, PPD: Postpartum depression; a) Association between prevalent depression (both old and new cases of depression) and average particulate air pollution (PM_2.5_); b) Association between chronic PPD (mothers that had depression during pregnancy and continued to be depressed at 6 months postpartum) and average particulate air pollution (PM_2.5_); c) Association between new-onset PPD at 6 months postpartum and average particulate air pollution (PM_2.5_).

We assessed the association between PPD and average PM_2.5_ exposure during pregnancy for EPDS ≥ 10, EPDS ≥ 12, and EPDS ≥ 13) (Fig. 1). The risk of prevalent PPD and new-onset PPD (EPDS ≥ 13) increased by 49% (RR: 1.49, 95%CI: 1.02–2.19) (Fig. 1a), and 167% (RR: 2.67, 95%CI: 1.37–5.21) (Fig. 1c), respectively, per 5-μg/m^3^ increase in average PM_2.5_ during pregnancy, similar to analyses performed in the larger cohort of 509 women (24). Likewise, there was a positive association between the average PM_2.5_ exposure during pregnancy and the risk of new-onset PPD using an EPDS ≥ 10 (RR: 2.12, 95%CI: 1.28–3.54) and an EPDS ≥ 12 (RR: 2.07, 95%CI: 1.10–3.91) (Fig. 1c), respectively. No association was found between the average PM_2.5_ and chronic PPD for all cut-offs (Fig. 1b).

**Figure 2:**
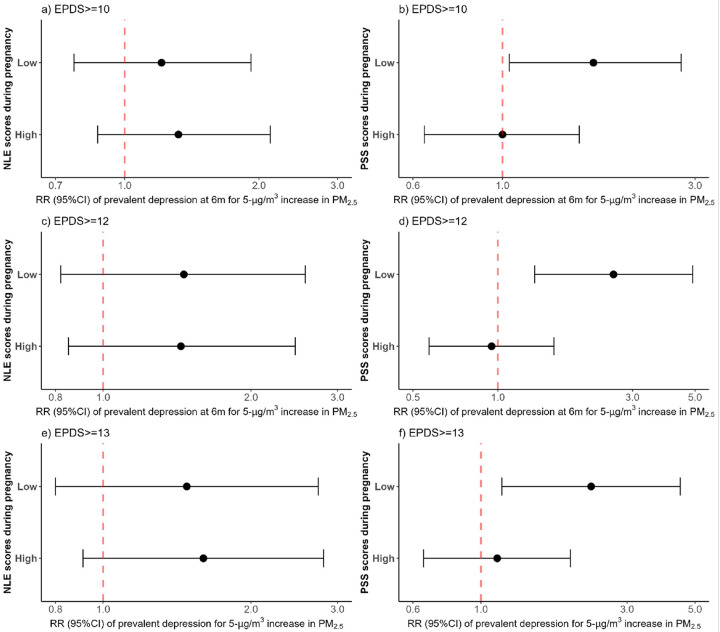
Association between prevalent depression and average particulate air pollution (PM_2.5_) exposure during pregnancy according to negative life events and perceived stress scores stratum. Analyses were adjusted for maternal age, maternal education, socioeconomic status, body mass index, maternal exposure to smoke inside the home, and gestational age at birth. NLE: Negative life events, PSS: Perceived stress scale score. EPDS: Edinburgh Postnatal Depression Scale, PPD: Postpartum depression; Association between prevalent PPD at 6 months postpartum and average particulate air pollution (PM_2.5_) according to NLE stratum for a) EPDS≥10, c) EPDS≥12, and e) EPDS≥13. Association between prevalent PPD at 6 months postpartum and average particulate air pollution (PM_2.5_) according to PSS stratum for b) EPDS≥10, d) EPDS≥12, and f) EPDS≥13

We then investigated how prenatal stress modified the relationship between PM_2.5_ and prevalent PPD. Perceived stress modified the association between PM_2.5_ exposure and prevalent PPD, but not NLE (Fig. 2). Each 5-μg/m^3^ increase in pregnancy PM_2.5_ exposure was associated with 129% increased risk of prevalent PPD (EPDS ≥ 13) in mothers with low PSS (RR: 2.29, 95%CI: 1.17–4.47) (Fig. 2f). Similarly, there was an increased risk of prevalent PPD per 5-μg/m^3^ increase in average PM_2.5_ among mothers with low PSS stratum using an EPDS ≥ 10 or EPDS ≥ 12 (RR: 1.68, 95%CI: 1.04–2.77; RR: 2.57, 95%CI: 1.35–4.90, respectively) (Fig. 2b and 2d).

The analyses between chronic PPD and average PM_2.5_ exposure during pregnancy according to NLE and PSS stratums did not yield any significant association (Supplementary Fig. 1a-1f). However, the effect of PM_2.5_ on chronic PPD appeared to be in the positive direction among mothers with low PSS across the different EPDS threshold (Supplementary Fig. 1b, 1d, and 1f), consistent with the above findings for prevalent PPD.

**Figure 3:**
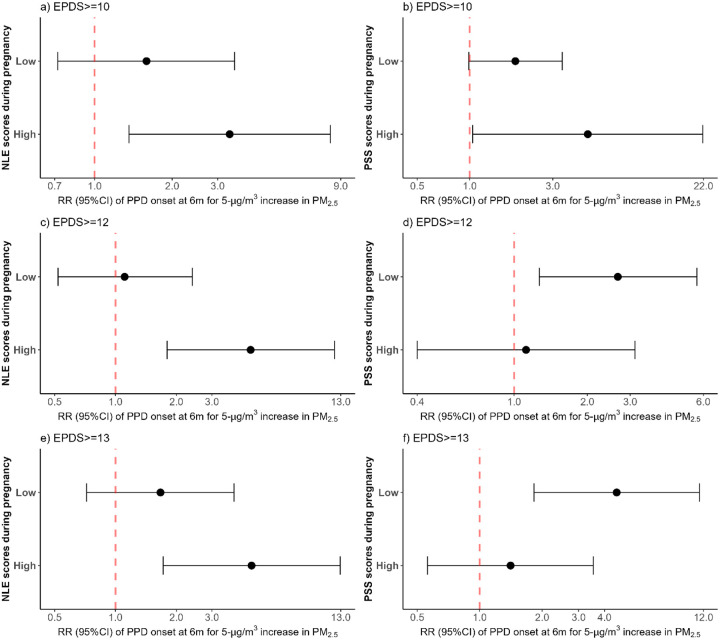
Association between postpartum depression onset at 6 months postpartum and average particulate air pollution (PM_2.5_) exposure during pregnancy according to negative life events and perceived stress scores stratum. Analyses were adjusted for maternal age, maternal education, socioeconomic status, body mass index, maternal exposure to smoke inside the home, and gestational age at birth. NLE: Negative life events, PSS: Perceived stress scale score. EPDS: Edinburgh Postnatal Depression Scale, PPD: Postpartum depression; Association between new-onset PPD at 6 months postpartum and average particulate air pollution (PM_2.5_) according to NLE stratum for a) EPDS≥10, c) EPDS≥12, and e) EPDS≥13. Association between new-onset PPD at 6 months postpartum and average particulate air pollution (PM_2.5_) according to PSS stratum for b) EPDS≥10, d) EPDS≥12, and f) EPDS≥13.

The associations between PM_2.5_ exposure and new-onset PPD according to NLE and PSS stratum were assessed (Fig. 3). Each 5-μg/m^3^ increase in average PM_2.5_ during pregnancy was associated with a 371% increase in the risk of new-onset PPD (EPDS ≥ 13) in mothers with high NLE (RR: 4.71, 95%CI: 1.72–12.92) (Fig. 3e) and a 358% increase among those with a low PSS scores (RR: 4.58, 95%CI: 1.83–11.49) (Fig. 3f). Similar findings were observed using an EPDS ≥ 12 in mothers with high NLE (RR: 4.66, 95%CI: 1.80–12.10) Fig. 3c) and among those with low PSS (RR: 2.67, 95%CI: 1.27–5.64) (Fig. 3d). Increased risk of new-onset PPD per 5-μg/m^3^ increase in average PM_2.5_ was also observed among mothers with high NLE (RR: 3.34, 95%CI: 1.36–8.21) (Fig. 3a), and with high PSS (RR: 4.77, 95%CI: 1.04–21.81) using an EPDS ≥ 10, although the association was borderline among those with a low PSS (RR: 1.83, 95%CI: 0.99–3.40) (Fig. 3b).

Finally, we assessed whether the association between average PM_2.5_ level during pregnancy and depression depended on stress (Supplementary Table 1). Indeed, a statistically significant interaction was observed between the average PM_2.5_ level and PSS on new-onset PPD (p = 0.035) using an EPDS ≥ 13 as well as between average PM_2.5_ level and NLE on new-onset PPD o using an EPDS ≥ 12 (P = 0.005). A similar finding was observed between average PM_2.5_ level and PSS on prevalent PPD using an EPDS ≥ 12 (Supplementary Table 1).

## Discussion

This study assessed the modifying role of prenatal psychosocial stress on the association between prenatal PM_2.5_ exposure and postpartum depression in Mexico, using three EPDS cut-offs (≥ 13, ≥ 12, and ≥ 10). We observed that PM_2.5_ exposure during pregnancy was consistently associated with increased risk of new-onset PPD across all thresholds. Among mothers with a low PSS, the effect of PM_2.5_ on prevalent PPD was stronger independently of the EPDS threshold used. Similar findings were observed for new-onset PPD among mothers with a high NLE and a low PSS across all the three EPDS cut-offs. These findings indicate that PM_2.5_ exposure during pregnancy is a consistent environmental risk factor for PPD, independently of the EPDS threshold used. Whether using a lower threshold (EPDS ≥ 10) to capture milder symptoms or a higher cut-off to identify cases with more marked symptomatology, the association between PM_2.5_ and new-onset PPD remained consistent. More importantly, the findings indicate that prenatal stress, particularly high exposure to NLE and low PSS may heighten the effect of ambient air pollution on new-onset PPD at 6 months postpartum, regardless of the cut-off used. However, we observed no statistically significant association between depression that began during pregnancy and persisted to six months postpartum (i.e., chronic PPD), suggesting that PM_2.5_ may not play a significant role in the continuation of pre-existing depressive symptoms. The fact that PM_2.5_ was positively associated with prevalent PPD which included both new and old cases of probable depression suggests that PM_2.5_ may contribute more to the incidence of new cases or exacerbate existing symptoms enough to meet the minimal EPDS threshold, which was evident when considering new-onset PPD, which includes only new cases of PPD at 6 months postpartum.

The amplified effect of PM_2.5_ on prevalent depression, particularly on new-onset PPD in mothers with a low PSS highlights the increase vulnerability of these mothers to the mental health effects of PM_2.5_. In this population, mothers with low perceived stress might not have established coping mechanisms, and therefore, be more overwhelmed by environmental stressors such as air pollution. Similarly, these mothers may live in environments with greater environmental hazards due to socioeconomics challenges and lack social support networks making them even more vulnerable to the effect of PM_2.5_. As shown in previous studies, exposure to higher levels of PM_2.5_ may elicit a more pronounced biological stress response in these mothers, which may lead to over-activation of the HPA axis (50–52), elevated cortisol (52, 53), and release of pro-inflammatory cytokines like 1α (IL-1α), IL-1β, IL-6, and tumor necrosis factor-α(17, 52, 53), with subsequent downstream consequences on maternal mental health. Given that perceived stress is subjective, mothers with low PSS can still face significant stressors like PM_2.5_ and other postpartum life challenges that make them more susceptible to the risk of these outcomes, if effective coping mechanisms or social support is lacking (54, 55). In this study, the NLE captured events that occurred in the past six months, while the PSS focused on stress that occurred in the last month during the third trimester of pregnancy. Although the NLE and PSS capture different dimensions of stress, it is possible that stressful events throughout the gestational period align more closely to the detrimental effect of PM_2.5_ on new-onset PPD at six months postpartum, compared to perceived stress. The stronger effect of PM_2.5_ on the risk of new-onset PPD in mothers with high NLE scores across all three threshold suggests that prolonged exposure to stressors like NLE may increase their susceptibility to air pollution, increasing the risk of new-onset PPD at 6 months. However, we observed that the effect of PM_2.5_ on prevalent PPD at 6 months remained similar across different levels of NLE scores indicating that NLE do not act as an effect modifier in this relationship. The difference in the effect of PM_2.5_ on prevalent PPD and new-onset PPD across NLE scores suggests that the combination of air pollution and NLE has a stronger influence on the later postpartum onset of depression rather than on depression that is persistence from pregnancy to 6 months postpartum. Overall, our findings highlight the interplay between environmental and psychosocial stressors, emphasizing pregnancy as a heightened period of susceptibility for postpartum mental health outcomes. This also highlights the need to account for air pollution and the need for well-characterized prenatal stressors when assessing the risk to postpartum maternal mental health outcomes.

While past analyses established the independent link between both prenatal stress (6, 13–15)and PM_2.5 (21, 22)_ with PPD, there is a lack of studies assessing the role of prenatal stress on the association between prenatal PM_2.5_ exposure and the risk of PPD. Given that both increased PM_2.5_ and prenatal stress independently place significant psychological strain on individuals (18), primarily by activating the HPA axis and triggering an inflammatory response, such disruption in the presence of the combined effect of prenatal stress and PM_2.5_ may overwhelm stress regulation mechanisms, exacerbating the risk of PPD particularly new-onset PPD at 6 months as we see here.

Our study has strengths and limitations. To our knowledge, this is the first study to examine whether the association between PM_2.5_ and the risk of PPD differs according to prenatal stress levels. We used an EPDS ≥ 13 considered as a conservative threshold to identify postpartum women at risk for major depressive symptoms (33, 36), and compared our findings with two other proposed thresholds (EPDS ≥ 10, EPDS ≥ 12) for screening PPD in Mexico to determine robustness of the associations. Data used in this study came from a well-defined longitudinal cohort study, with substantial sample size, and high spatial resolution of PM_2.5_ exposure data that covers the whole pregnancy period. The study showed that prenatal stress particularly low PSS and high NLE exacerbated the effect of prenatal PM_2.5_ exposure on PPD, especially new-onset PPD. However, our findings must be interpreted as an increased risk for PPD as the EPDS is a screening tool used to assess possible depression, instead of clinical depression per se. Furthermore, considering that the NLE scale and the PSS used in this study represent different periods of exposure during pregnancy, the results of our study must be interpreted accordingly. It is necessary to measure perceived stress throughout all trimesters of pregnancy for comparative purposes and to identify critical periods of vulnerability during pregnancy.

## Conclusion and recommendation

Prenatal low perceived stress and high negative life events exacerbate the effect of prenatal PM_2.5_ exposure on the risk of PPD, particularly new-onset PPD, across EPDS thresholds of ≥ 10 to ≥ 13. This highlights the robustness of our findings and the complex interplay between environmental and psychosocial factors, particularly among mothers facing psychosocial challenges. More longitudinal study is needed particularly using perceived stress collected across all trimesters of pregnancy to understand the interplay between environmental exposures and prenatal stress on PPD.

## Supplementary Material

Supplementary Files

This is a list of supplementary files associated with this preprint. Click to download.

- Supplementarymaterials.pdf

## Figures and Tables

**Table 1:** Characteristics of the study population (N = 475).

Variables	Description N (%)
Maternal age (years) during pregnancy	27.7 (SD: 5.5)
Maternal exposure to smoke inside home during pregnancy	
Yes	145 (30.5)
No	330 (69.5)
Maternal education during pregnancy	
<High school	195 (41.1)
High school	169 (35.6)
>High school	111 (23.4)
Socioeconomic status during pregnancy	
Low	244 (51.4)
Medium	180 (37.9)
High	51 (10.7)
Average PM2.5 in pregnancy	22.9 (IQR: 20.3–24.5)
Mother’s body mass index pre-pregnancy	26.9 (SD: 4.1)
NLE during pregnancy	3 (IQR:2–5)
Perceived stress during pregnancy	5 (IQR: 3–7)
Gestational age at birth (weeks)	39 (IQR: 38–39)
EPDS at 6 months postpartum	5 (IQR: 2–10)
EPDS ≥ 10 prevalent PPD	
Yes	144 (30.3)
No	331 (69.7)
EPDS ≥10 chronic PPD	
Yes	99 (20.80)
No	376 (79.20)
EPDS < 10 new-onset PPD	
Yes	45 (9.47)
No	430 (90.53)
EPDS ≥ 12 prevalent PPD	
Yes	98 (20.63)
No	377 (79.37)
EPDS ≥ 12 chronic PPD	
Yes	55 (11.60)
No	420 (88.40)
EPDS < 12 new-onset PPD	
Yes	43 (9.05)
No	432 (90.95)
EPDS ≥ 13 prevalent PPD	
Yes	86 (18.1)
No	389 (81.9)
EPDS ≥ 13 chronic PPD	
Yes	46 (9.70)
No	429 (90.30)
EPDS < 13 new-onset PPD	
Yes	41 (8.6)
No	434 (91.4)

EPDS: Edinburgh Postnatal Depression Scale, NLE: Negative life events, SD: Standard deviation, IQR: Interquartile range, prevalent PPD: all participants with EPDS equal to or above the cut-off at 6 months, regardless of EPDS score during pregnancy; chronic depression: EPDS equal to or above cut-off during pregnancy and at 6 months postpartum; new-onset PPD: EPDS equal to or above cut-off at 6 months postpartum, but below the cut-off during pregnancy

## Data Availability

The data that support the findings of this study are available from the corresponding authors upon reasonable request.

## Abbreviations

AOD: Aerosol Optical Depth
BMI: Body mass index (BMI)
CRH: Corticotropin-releasing hormone
CRISYS: Crisis in Family Systems-Revised
EPDS: Edinburgh Postnatal Depression Scale
HPA: Hypothalamic-pituitary-adrenal
IMSS: Instituto Mexicano del Seguro Social
MODIS: Moderate Resolution Imaging Spectroradiometer
NLE: Negative life events
PM2.5: Ambient air pollution
PPD: Postpartum depression
PROGRESS: Programming Research in Obesity, Growth, Environment, and Social Stressors
PSS: Perceived stress score
SES: Socioeconomic status
